# Effect of Maternal Water Restriction on Sexual Behavior, Reproductive Performance, and Reproductive Hormones of Male Rat Offspring

**DOI:** 10.3390/ani10030379

**Published:** 2020-02-26

**Authors:** Ja’far Al-Khaza’leh, Rami Kridli, Belal Obeidat, Shahera Zaitoun, Anas Abdelqader

**Affiliations:** 1 Faculty of Agricultural Technology, Al-Balqa Applied University, P.O. Box 19117, Al- Salt, Jordan; zaitoun@bau.edu.jo; 2 New-Life Mills, A Division of Parrish & Heimbecker, Limited, Cambridge, ON N1T 2H9, Canada; rkridli@newlifemills.com; 3 Faculty of Agriculture, Jordan University of Science and Technology, Irbid 22110, Jordan; bobeidat@just.edu.jo; 4 School of Agriculture, The University of Jordan, Amman 11942, Jordan; a.abdelqader@ju.edu.jo

**Keywords:** maternal stress, water, pregnancy outcomes, rat offspring, Sprague–Dawley rats

## Abstract

**Simple Summary:**

Drinking water restriction that a pregnant female may encounter is one of the major stressors that could affect pregnancy outcome and understanding its effects is important in animal welfare concerns and pregnancy outcomes for women or animals. To the best of our knowledge, maternal drinking water restriction has not yet been investigated in previous studies as a potential factor affecting reproductive performance of male rat offspring over first and second generations. Therefore, we aimed to assess the consequences of maternal drinking water restriction stress on sexual behavior, reproductive performance, and reproductive hormones of male rat offspring. The 50% maternal water restriction during the second half of pregnancy reduced body weights of rat dams and offspring at birth and negatively impacted some reproductive characteristics. However, reproductive performance and hormones of males were not adversely affected.

**Abstract:**

The present study aimed to investigate the effect of maternal water restriction on sexual behavior, reproductive performance, and reproductive hormones of male rat offspring. Forty pregnant female rats were divided into two equal groups: Control (C) and water-restricted (WR). Control dams had ad libitum water access throughout pregnancy, while dams in the WR group were subjected to 50% water-restriction from day 10 of pregnancy onwards. The maternal water restriction provoked a significant reduction (*p* < 0.05) in body weight of dams before delivery and at birth and litter body weights of offspring at birth. Maternal water restriction did not affect relative weights of reproductive and body organs of male rat offspring. All hormonal concentrations, sperm count, and vitality in male rat offspring were not significantly affected by maternal water restriction. Maternal water restriction exposure induced significant (*p* < 0.05) reduction in intromission latency, intromission frequency, and post-ejaculation interval in male rat offspring while a significant (*p* < 0.05) increase in the ejaculation latency was detected in maternal WR group. In conclusion, this study suggests that maternal water restriction had a negative impact on some reproductive characteristics but did not severely affect reproductive performance and reproductive hormones of male rat offspring.

## 1. Introduction

Various experimental studies conducted on mice, rats, and pigs showed that many environmental and ethological prenatal stressors can have injurious effects on the pregnancy and early and long-term adverse effects on the offspring. Different prenatal treatments and stress types, such as medications [1], drinking water restriction and deprivation [2,3,4], feed restriction [5], immobilization [6,7], light intensity [8], stocking density [9], restraint or social stressors [10,11,12,13,14,15,16], heat or noise [17], to which a pregnant female is exposed, can affect pregnancy outcome and influence many aspects of physiological systems in the offspring including sexual behavior, puberty onset, gonad function, reproductive hormones, and development of the reproductive organs.

The detrimental consequences of maternal stress exposure depend on type of stress, its frequency and severity, pregnancy stage, and sex offspring [18]. Water is required for all vital functions in the body [19,20]. It is the major component of the animal’s live body weight; 50% to 80% of its body weight [21,22]. Water is needed to flush out wastes, aid in digestion, and ensure the body absorbs the essential nutrients from the food [23]. It also helps to restore and revitalize body. During pregnancy, these essential functions are even more important; water is an essential and critical nutrient required in sufficient amounts to cope with the demands of ever-changing bodies and maintain a healthy environment for the fetus [24]. Exposure to stress during pregnancy is associated with a variety of alterations in male offspring. Maternal under nutrition during pregnancy can cause long-lasting effects on the health of the offspring [25,26].

Studies reported differences between male and female fetuses’ response to the maternal environment. A review by Sandman et al. [27] showed that growing male fetuses are more vulnerable to effects during pregnancy than female fetuses. Moreover, evidence from Ashworth et al. [14] indicates that the fetal reproductive axis of male pigs carried by sows that were stressed is more susceptible to environmental changes than that of female siblings. Furthermore, the developing male reproductive axis is more responsive to maternal stress in that prenatal stress may compromise aspects of male reproductive development compared with control males [10]. The mechanism that shows transmission of the physiological effects of maternal stressors to developing fetuses was clarified by Brunton [18] and Goncharova [28]. It includes modification in the responsiveness of the maternal and offspring hypothalamic–pituitary–adrenal (HPA) axes [18,29,30,31]. However, whether or not a maternal water restriction is comparable to the types of social stresses in respect to its effects on the HPA axes remains unknown.

The hypothesis of the current study was that the exposure to drinking water restriction imposed during pregnancy could have adverse consequences on offspring development, namely fertility, sexual behavior, reproductive performance, and reproductive hormones. Understanding the effects of maternal drinking water restriction is important in animal welfare concerns and pregnancy outcomes for women or animals. Maternal drinking water restriction has not yet been investigated in previous studies as a potential factor affecting reproductive performance of male rat offspring over first and second generations. The present study, therefore, aims to assess the consequences of maternal drinking water restriction stress on sexual behavior, reproductive performance, and reproductive hormones of male rat offspring.

## 2. Materials and Methods

### 2.1. Animals

In this study, 40 virgin female albino Sprague-Dawley rats weighing on average 190.3 ± 15.7 g and aged 9 weeks old were used. The female rats were obtained and raised in the Animal House Unit at Jordan University of Science and Technology (JUST) between January and March 2018. All animal care protocols and experimental procedures were approved by the Animal Care and Use Committee at JUST (Approval #: 16/3/3/146) and were in accordance with the National Institute of Health on the use and care of laboratory animals (USA) Guidelines. Animals were housed separately in polypropylene cages and maintained under standard laboratory conditions (ambient temperature of 22 ± 2 °C, 12/12 h light-dark cycle). The animals had access to standard chow and water ad libitum and were allowed to adapt for one week before beginning the experiments.

Initial and final body weights were measured by using a weighing scale. Each female with regular estrous cycle was introduced overnight and mated with a proven sexually experienced fertile male weighing on average 309.6 ± 23.6 g at ratio of one male to two females to induce pregnancy. In the following morning, the beginning of pregnancy was confirmed by sperm presence as vaginal smear (white sperm plug) and its positive presence was designated as day zero (D0) of gestation.

### 2.2. Experimental Design

*Animal grouping:* Pregnant female rats were randomly assigned into one of two groups of 20 rats each as follows: The control group (C, *n* = 20, with 100% ad libitum free water access) and experimental water-restricted group (WR, *n* = 20, with 50% water restriction). Normal daily water intake for a 24-h period (100% ad libitum free access) was quantified during the pre-pregnancy period by measuring the daily water intake of each dam and calculating a group mean.

**Figure 1:**
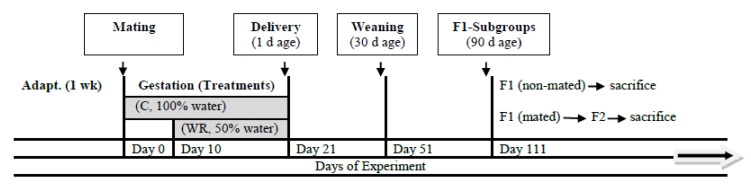
Experimental design.

*Water restriction application*: Dams from C group had ad libitum free access to distilled water from conception until they gave birth, while stressed dams in WR group were exposed to water restriction by providing only half of daily water requirement (50% restriction) from day 10 of pregnancy onwards. After delivery, the two groups were given free access to water and food. Makhmudov et al. [32] adopted a 50% water and feed restriction protocol in Wister rats from the outset of pregnancy. A similar protocol was conducted by Mansano et al. [3] on Sprague–Dawley rats from day 10 of pregnancy onwards. Maternal body weight of all dams from two groups was recorded weekly until delivery. Figure 1 illustrates the experimental design.

### 2.3. Pregnancy Outcomes and Sexual Behavior Assessment

Table 1 summarizes the outcome after delivery of dams. Following delivery, pups of the first generation (F1) were sexed on postnatal day 1 and kept with their mothers until weaning on postnatal day 21.

The birth weights, litter size, and survival index of pups were assessed. Then, male pups only were kept separately and weighed weekly until the age of 90 days. Consequently, a subset of two male offspring from each litter of dam were randomly selected and divided into two subgroups. The first subgroup (non-mated) consisting of male offspring, which were anesthetized and humanely killed (rats were anesthetized using intraperitoneal injection of Ketamine-Xylazine combination (using 1 mL syringe, 75–90 mg/kg ketamine and 5–10 mg/kg xylazine in the same syringe) followed by cardiac puncture for blood collection to assess hormone concentrations. The second subgroup (mated) consisted of male offspring, which was mated to produce the second generation (F2) and was monitored to assess its sexual behavior.

For the second subgroup, each adult male offspring (F1) from each group (C, and WR groups) weighing on average 232 ± 28 g was mated with a regular estrous cycle female from an additional cohort of non-treated animals with one to one mating ratio. Male (F1) was housed individually in the observation chamber (a clear acrylic box) and was allowed to adapt for 10–15 min. Then, a sexually receptive female was introduced into the chamber and the sexual behavior of the male was recorded for 30 min in a dark room using a tracking video camera. Due to financial limitations and for better control and more accurate observations, only 12 male rats from each group were randomly assigned for sexual behavior. The following sexual behavior parameters of male offspring rats (F1) were measured according to Mohamed et al. [33]: (1) Mount latency: Time from introduction of the female until the first mount; (2) mount frequency: Number of mounts preceding ejaculation; (3) intromission latency: Time from introduction of the female until the first intromission (vaginal penetration); (4) intromission frequency: Number of intromissions preceding ejaculation; (5) ejaculation latency: Time from the first intromission until ejaculation; (6) ejaculation frequency: The number of ejaculations within 30 min; (7) post-ejaculatory interval: Time from ejaculation until the next intromission. After observing the sexual behavior, a vaginal smear was carried out to examine for the existence of spermatozoa ensuring that mating had taken place and appointed it as day zero (D0) of pregnancy. In addition to sexual behavior assessment, the productive performance of male rat offspring (F1) of the second subgroup was also evaluated by assessing some of reproductive parameters of their offspring (F2) such as litter size and weight.

### 2.4. Reproductive and Body Organ Weights

A week after sexual behavior assessment, male rat offspring (F1) of the second subgroup were also anesthetized and humanely killed. Laparotomy was then immediately performed, and the internal reproductive organs (testes, seminal vesicles, prostate, epididymides, and vasa deferentia) were removed and then weighed after clearing the visible fats and connective tissues. Additionally, different organs, namely the heart, liver, lungs, spleen, and kidneys, were carefully dissected and weighed in grams (absolute organ weight). Relative organ weight was calculated as = Absolute organ weight (g)/Body weight of rat on sacrifice day (g) × 100.

### 2.5. Blood Sampling and Hormones Assessment

Trunk blood samples were taken by intra-cardiac puncture of sacrificed animals and collected from each animal into anticoagulated test tube using 5% EDTA. Blood samples were centrifuged at 3000 RPM for 30 min. The separated plasma was stored frozen at −20 °C for subsequent analysis of serum hormones. Serum levels of hormones (cortisol, testosterone, thyroid-stimulating hormone (TSH), and luteinizing hormone (LH)) were measured in duplicate using enzyme-linked immunosorbent assay (CORTISOL ELISA, REF: DKO001; FREE TESTOSTERONE ELISA, REF: DKO015; Rat TSH ELISA KIT, CK-E30271; Rat LH ELISA KIT, CK-E30447).

### 2.6. Assessment of Semen Quality

For sperm parameters evaluation, semen was gently squeezed out of the seminal vesicles and epididymis, and vas deferens were dissected. Determination of total epididymal concentration was performed by regular counting protocol using a heamocytometer. Total epididymal sperm collection was performed by suspension of the right epididymis into 1 mL of buffered formal saline solution as described by Wang [34]. A drop of semen was taken prior to the collection by slight squeezing for smear preparation. Smears were prepared using eosin-nigrosine one-step staining procedure on clean slides and kept for later analysis. The percentage of sperm vitality was determined by counting the proportion of stained (dead) sperms in 5 fields in each slide (about 400 sperm). Testis was transferred into bottles containing 10% formalin for later histological analysis.

### 2.7. Statistical Analyses

**Figure 2:**
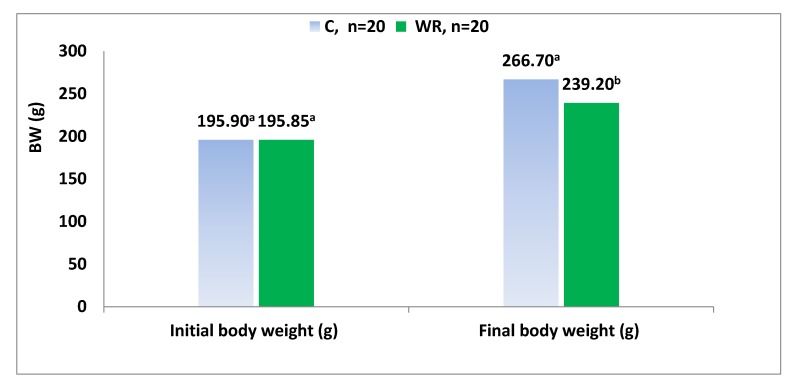
Initial and final body weights of pregnant dams in each group. Bars marked with different letters differ significantly (*p* < 0.05); values that are marked with the same letter are not significantly different.

**Figure 3:**
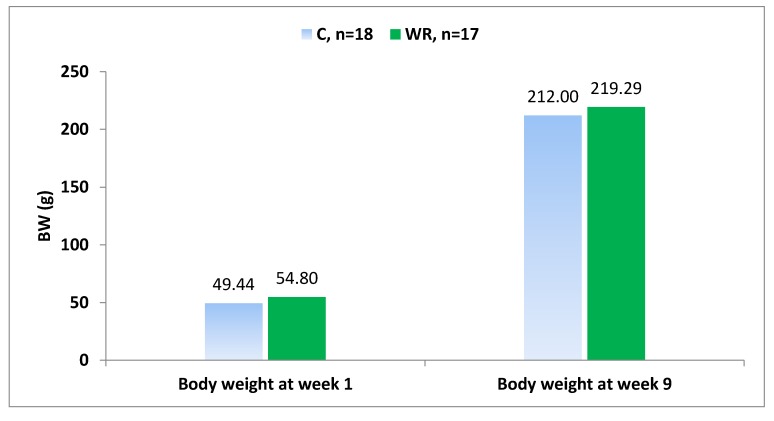
Body weight of male offspring (F1) post weaning in control and water-restricted group. SE: Standard error of the mean. Differences (*p* < 0.05) were not detected.

The data were analyzed using a general linear model (GLM) procedure of SAS software 9.3 [35]. Continuous variables were regarded as dependent variables while treatments were fitted as explanatory variable. The data were tested for normality and homogeneity of error variances prior to model fitting to get a normal distribution for the residuals. After assessing normality and homogeneity, the data with normal distribution and homogenous variances were analyzed to investigate significant differences between treatment groups. In Figure 2, the dam was considered the experimental unit of study in the statistical analyses, whereas in Figure 3 and in Table 2, Table 3, Table 4, Table 5, Table 6 and Table 7, the offspring was considered the experimental unit of study in the statistical analyses. All comparisons of continuous variables between control and stressed animals with unbalanced data presented as means ± standard error (SE) and *p*-value of < 0.05 was considered as statistically significant.

## 3. Results

### 3.1. Body Weights and Pregnancy Outcomes

Changes in dams’ body weights before parturition are shown in Figure 2. The mean weight losses relative to C values after 9 days of water restriction (from day 10 of pregnancy onwards) were approximately 29% for the WR group.

Pregnancy outcomes and characteristics of F1 are presented in Table 2. The average body weights of dams following parturition and average pup body weights of rat offspring at birth were significantly higher in the C group compared to the WR group. However, no significant differences were found in the length of dam gestation, litter size at birth and at weaning, and pup body weights at weaning between C and WR animals (Table 2). The % 30-day survival index (SI) of pups at weaning was similar between the C and WR groups. The % of dead pups at weaning was similar between the C and WR groups. In general, male pups outnumbered female pups by a ratio of 1.1 to 1 and comprised about 48.00% and 55.42% of the total litter size in the C and WR groups, respectively.

Figure 3 shows that the body weights of all male offspring (F1) post-weaning increased significantly with increasing age of animals. However, the body weights of males (F1) post weaning at weeks 1 and 9 were similar between C and WR groups (49.44 ± 2.4 vs. 54.80 ± 2.5) and (212.00 ± 7.5 vs. 219.29 ± 7.7), respectively.

### 3.2. Reproductive and Body Organ Weights of F1 Male Rat Offspring

The relative organ weights of different reproductive and body organs are shown in Table 3. There were no differences (*p* > 0.05) in absolute and relative weights of reproductive organs (testes, epididymis, vas deferentia) and other organs (kidney, liver, spleen) of F1 mated male rat offspring between two groups.

### 3.3. Reproductive Hormones of F1 Male Rat Offspring

Mean serum cortisol, LH, testosterone, and TSH levels of two groups for mated and non-mated F1 male rat offspring levels are presented in Table 4. All hormonal concentrations were not different (*p* > 0.05) between the two groups in mated and non-mated subgroups with the exception of LH of non-mated subgroups. LH level of non-mated male rat offspring was higher (*p* < 0.05) in C group compared to WR group.

### 3.4. Sperm Parameters of F1 Male Rat Offspring

Sperm counts were similar between C group and WR group (Table 5). Observation on sperm vitality of F1 male rat offspring indicated no significant differences between the C and WR groups.

### 3.5. Sexual Behavior of F1 Male Rat Offspring

The effect of maternal water restriction on the sexual behavior of F1 male rat offspring is shown in Table 6. Treatment with water restriction induced reductions (*p* < 0.05) in intromission latency, intromission frequency, and post-ejaculation interval in comparison with corresponding values of control rats. Mount latency tended to be lower (*p* = 0.0602) in the WR group compared to the C group. However, an increase (*p* < 0.05) in the ejaculation latency was detected in the WR group compared with the C group.

### 3.6. Reproductive Performance of F1 Male Rat Offspring

The average body weights of non-treated dams at birth, litter body weights, and litter size of F2 rat offspring at birth were similar between C and WR groups (Table 7).

## 4. Discussion

In the present study, one dam died, and two dams gave stillbirth at the end of gestation period in the WR group. These losses in WR group, and the lack of such losses in the C group, could confirm the exhausting impact of water restriction during the critical stage of pregnancy on dams. The gestation length of dams in this study were not affected by water restriction. Regardless of stress type, a previous study by Guan et al. [36] showed that the gestation length of dam Sprague–Dawley rats was not affected by maternal water deprivation for three days at late gestation. Another study by Alwasel [10] also showed that the gestation length of dam Wistar rats was not impacted by maternal food restriction. The body weights of stressed pregnant dams were significantly affected by water restriction during the last stages of pregnancy compared to control pregnant dams. This can be ascribed to reduction in food intake by dams. A study by Bekkevold et al. [37] showed that food intake was significantly reduced in 50% and 75% water-restricted and water-deprived groups of mice and caused weight losses compared to control groups. Mansano et al. [3] reported that 50% of water restriction during the late stage of pregnancy of Sprague–Dawley rats resulted in about 25% less food intake than controls. Body weight and food intake were also significantly reduced in Sprague–Dawley pregnant and non-pregnant rats that were exposed to water deprivation for 48 h [4]. This reduction in body weights of stressed pregnant dams during pregnancy was confirmed by lower body weights of litter at birth than that in control animals. However, the body weights of litter at weaning and their survival index, and the body weight of males (F1) post-weaning at week 1 and 9 were similar between two groups. This can be attributed to the fact that catch up growth of WR to C occurred and the F1 rat offspring recovered from maternal water restriction effects after providing water and feed ad libitum. A study by Mansano et al. [3] showed that there was no difference in litter size or pup survival in rat offspring born to mothers exposed to 50% water restriction during pregnancy. The same study showed that the body weights of rat offspring at birth were smaller in 50% water-restricted group compared to control, but this difference was no longer seen prior weaning due to occurrence of catch-up growth.

In this study, water restriction during pregnancy did not cause any adverse effect on reproductive or organ weights of F1 male rat offspring compared to controls. The relative organ weights investigated in this study were slightly higher than normal values reported by Alemán et al. [38]. This could also indicate that the normal physiological functions of those organs were not negatively impacted by maternal water restriction. Due to fact that the body weights of pups at birth (day 1) were affected by maternal water restriction, differences were expected between C and WR groups regarding the absolute and relative weights of reproductive and body organs of F1 male rat offspring. However, due to catch up growth of WR F1 male rat offspring, no difference in the absolute or relative body weight between groups was seen by 90 days of age. A study by Mansano et al. [3] showed no differences in the absolute and relative weights of kidney and liver of rat offspring at 21 days of age in 50% maternal water-restricted group compared to control. Chehreie et al. [2] found that a prenatal water deprivation for 48 h at the end of third trimester of gestation causes significant reduction in total body weight and the weight of testes of male pubertal Sprague–Dawley Rats. Other findings showed no alteration in weights of the testis and epididymis of male rats exposed to prenatal restraint stress [11,39]. However, differences in the type of stress exposed to the rats during pregnancy and its corresponding mechanism of action should be taken into consideration. In the present study, the normal weights of the testis and epididymis of male rat offspring in WR group could be explained by normal level of testosterone.

Animals respond to stressful challenges by modifying the hypothalamo-pituitary-adrenal axis and elevating the circulating levels of glucocorticoids [31]. However, evidence for effects of water restriction on glucocorticoid levels in pregnant rats has not been documented in studies. Thus, it is not possible based on present results to definitely establish whether this challenge is associated with modification of the HPA axis and glucocorticoid levels in the dams. In the present study, cortisol has been measured as an indicator of stress, yet corticosterone is the equivalent in the rat. Similarly, other studies have used cortisol as an index of stress in the rat [40,41,42]. A study by Gong et al. [43] showed that rodent serum cortisol and corticosterone were closely correlated in patterns under different physiological or stressful conditions.

In the present study, we did not measure circulating cortisol at an early age, shortly following exposure to prenatal maternal water restriction. When males were sacrificed at 111 days of age, serum cortisol concentrations, as a classic indicator of physiological stress, were not significantly higher in the WR group compared to the C group for mated and non-mated subgroups. Such lack of difference may suggest that the effect of maternal stress did not induce a chronic elevation of cortisol in the offspring. We might have seen a difference in cortisol concentrations had we tested at an earlier age. Moreover, the adaptation over time during pregnancy with this type of water restriction could also reduce the impact of water restriction as a stressor. A potential confirmation for this can be seen from the normal value of serum cortisol in the present study that was accompanied with normal level of LH, testosterone, and TSH of the male rat offspring of maternal WR group. The normal level of serum testosterone found in maternally stressed males seems to be the result of gonadal steroidogenesis due to synthesis and release of gonadotrophin-releasing hormone (GnRH), FSH, and LH hormones [31,44]. Our results are comparable with a study by Heiderstadt et al. [45] where water restriction to only 15 min every 24 h did not change corticosterone levels in male outbred rats. However, another study showed that a prenatal water deprivation for 48 h at the end of third trimester of gestation reduces the concentration of plasma testosterone of newborn male Sprague–Dawley rats [46]. According to a study by Pallarés et al. [13], serum LH and FSH levels were decreased at postnatal day 28 and testosterone was decreased at postnatal day 75 in prenatally restraint stressed Wistar rats offspring. Maternal stress chronically increased cortisol concentrations most likely reducing maternal circulating thyroid hormones [47]. In humans, glucocorticoid administration decreases plasma TSH levels and attenuates the pituitary TSH response to TRH stimulation [48]. In addition, glucocorticoids enhance the negative feedback effect of thyroid hormones on TSH release stimulation [48]. For the non-mated subgroup, the marked group difference in LH level could be ascribed to the negative feedback effect of testosterone secreted by the gonads of the maternally stressed males compared to maternally controlled males (7.93 vs. 3.90, Table 4) or it could be attributed to other factors that may be involved in the timing of LH surge (e.g., increase the responsiveness of pituitary gland to GnRH).

In the present study, maternal water restriction stress did not significantly reduce the sperm count and the percentage of sperm vitality in male rat offspring as shown in WR group. Similarly, testicular and epididymal weights and serum testosterone level in WR group were not significantly different from C group, which therefore could be the possible explanation for the normal sperm count and the percentage of sperm vitality in male rat offspring. Chehreie et al. [2] reported that prenatal water deprivation for 48 h at the end of the third trimester of gestation stress caused disruption in normal spermatogenesis of offspring and reduction in total sperm motility and progressive sperm motility in male pubertal Sprague–Dawley offspring rats. Such a difference between results of the present study and aforementioned study [2] could be attributed to differences in the type of stress applied, its frequency and severity, and timing of exposure during pregnancy period.

In order to evaluate the ability of male rat offspring of stressed pregnant mothers to successfully mate female rats and subsequently obtain viable offspring, sexual activity and performance of the F1 male offspring were assessed. Surprisingly, in the present study, maternal water restriction enhanced the sexual activity (libido) in male offspring rats as shown by mounting and intromission latency values in the WR group compared to the control group. The sexual activity of male rats could be explained by elevated testosterone level on the first day of interaction with estrous female before it falls back to the near level of control rats [49]. After the sexual motivation, intromission supposedly occurs, which is preceded by penile erection leading to ejaculation [50]. An improvement in sexual potency was also evidenced in maternal water restricted rats through decreased intromission frequency. Intromission frequency and ejaculation latency in this study were inversely proportional, as the number of intromissions increases the time from the first intromission until ejaculation decreases. Intromission latency time was shorter in the WR group, which indicates rats were more sexually active and had more desire but took longer ejaculation latency time compared to the C group. One explanation of that could refer to lower intromission frequency in the WR group than in the C group. Post-ejaculation interval was shorter in the WR group than in the C group and this can be explained by less effort and energy expended due to lower intromission frequency.

When comparing pregnancy outcomes of mating of F1 male rat from mothers exposed to 50% water restriction with non-treated females had ad libitum free access to water during pregnancy, it was observed that the average body weights of F2 non-treated mothers, litter body weights, and size of F2 rat offspring at birth were similar between C and WR groups. It seems that effect of maternal water restriction also did not pass to the second generation (F2). In the present study, normal sperm count and the percentage of sperm vitality of F1 male rat offspring might explain the normal litter size in the second generation (F2) of WR group.

## 5. Conclusions

The present study revealed that 50% maternal water restriction during the second half of pregnancy caused a significant reduction in body weights of mothers before delivery and at birth and litter body weights of F1 offspring at birth. However, dam gestation length, litter size at birth and at weaning, the percentage 30-day SI, and litter weight of pups at weaning and post-weaning of male rat offspring was not significantly affected. In addition, maternal water restriction did not adversely affect relative weights of reproductive and body organs, hormonal concentrations, sperm count and vitality, and sexual libido and performance of F1 male rat offspring.

## Figures and Tables

**Table 1:** Summary related to dams at the end of gestation period in each experimental group.

Variables	Groups	
	C	WR
	(*n* = 20)	(*n* = 20)
Non-pregnant dams (number)	2	0
Stillbirth dams (number)	0	2
Died dams at birth (number)	0	1

**Table 2:** Pregnancy outcomes and characteristics of F1 in control and water-restricted group.

Variables	Groups		Significance
	C	WR	
	Mean ± SE	Mean ± SE	
Gestation length (day)	20.94 ± 0.4	20.85 ± 0.0	ns
	(*n* = 18)	(*n* = 20)	
Dam wt at birth (g)	222.60 ± 3.6	205.25 ± 3.4	*
	(*n* = 18)	(*n* = 20) **	
Individual pup wt at birth (g)	5.99 ± 0.4	5.56 ± 0.4	*
	(*n* = 18)	(*n* = 20) **	
Litter size at birth (pup)	10.22 ± 0.5	9.75 ± 0.4	ns
	(*n* = 18)	(*n* = 20) **	
Individual pup wt at weaning (g)	33.42 ± 1.9	36.98 ± 1.98	ns
	(*n* = 18)	(*n* = 17)	
Litter size at weaning (pup)	9.66 ± 0.4	9.47 ± 0.4	ns
	(*n* = 18)	(*n* = 17)	
30-day SI of pups at weaning (%)	94.84 ± 2.0	95.98 ± 2.1	ns
	(*n* = 18)	(*n* = 17)	
Dead of pups at weaning (%)	00.56 ± 0.2	00.47 ± 0.2	ns
	(*n* = 18)	(*n* = 17)	
Male ratio at weaning (%)	48.00 ± 3.7	55.42 ± 3.8	ns
	(*n* = 18)	(*n* = 17)	
Female ratio at weaning (%)	52.00 ± 3.7	44.58 ± 3.8	ns
	(*n* = 18)	(*n* = 17)	
Sex ratio of pups (male: female)	1.1 ± 0.2	1.5 ± 0.2	ns
	(*n* = 18)	(*n* = 17)	

SE: Standard error of the mean, SI: Survival index. ** two litters that were stillborn, and one dam that died in the WR group were included in the analysis, ns: Not significant, * significant at *p* < 0.05.

**Table 3:** Relative weights (%) of reproductive and body organs of F1 male rat offspring in control and water-restricted group.

Variables	Groups	
	C	WR
	Mean ± SE	Mean ± SE
Testes	1.06 ± 0.1 (*n* = 18)	1.12 ± 0.1 (*n* = 15)
Epididymis and vas deferentia	0.69 ± 0.0 (*n* = 18)	0.71 ± 0.0 (*n* = 16)
Kidneys	0.75 ± 0.0 (*n* = 18)	0.83 ± 0.0 (*n* = 16)
Liver	3.61 ± 0.1 (*n* = 18)	3.65 ± 0.1 (*n* = 16)
Spleen	0.24 ± 0.0 (*n* = 18)	0.30 ± 0.0 (*n* = 16)

SE: standard error of the mean. Differences (*p* < 0.05) were not detected.

**Table 4:** Reproductive hormone levels of mated and non-mated F1 male rat offspring in each group.

Subgroup	Variables	Groups		
		C	WR	Significance
		Mean ± SE	Mean ± SE	
Mated	Cortisol (ng/mL)	1.56 ± 0.2	1.30 ± 0.2	ns
		(*n* = 18)	(*n* = 16)	
	LH (mIU/mL)	27.24 ± 0.4	26.49 ± 0.5	ns
		(*n* = 18)	(*n* = 16)	
	Free Testosterone (pg/mL)	8.26 ± 1.8	8.72 ± 1.9	ns
		(*n* = 18)	(*n* = 16)	
	TSH (mU/L)	11.29 ± 0.2	11.16 ± 0.3	ns
		(*n* = 18)	(*n* = 16)	
Non-mated	Cortisol (ng/mL)	0.72 ± 0.2	1.02 ± 0.2	ns
		(*n* = 18)	(*n* = 17)	
	LH (mIU/mL)	30.58 ± 0.6	28.67 ± 0.6	*
		(*n* = 18)	(*n* = 17)	
	Free Testosterone (pg/mL)	3.90 ± 1.4	7.93 ± 1.5	ns
		(*n* = 18)	(*n* = 16) **	
	TSH (mU/L)	12.12 ± 0.2	11.64 ± 0.2	ns
		(*n* = 18)	(*n* = 17)	

TSH: Thyroid stimulating hormone, LH: Luteinizing hormone, ns: Not significant, * significant at *p* < 0.05, ** One outlier with high deviation from the mean was removed for the analysis of testosterone to get a normal distribution for the residuals.

**Table 5:** Sperm parameters of F1 male offspring of control or water-restricted groups.

Variables	Groups	
	C	WR
	Mean ± SE	Mean ± SE
Sperm count (×10^6^ mL^−1^)	164.46 ± 19.1	167.20 ± 14.8
	(*n* = 18)	(*n* = 15)
Sperm vitality (%)		
Sperm live	91.86 ± 0.8	90.17 ± 0.9
	(*n* = 17)	(*n* = 15)
Sperm dead	9.14 ± 0.8	9.83 ± 0.9
	(*n* = 17)	(*n* = 15)

SE: standard error of the mean.

**Table 6:** Sexual behaviors of F1 male offspring of control or water-restricted groups.

Variables	Groups		Significance
	C	WR	
	Mean ± SE	Mean ± SE	
Mount latency (s)	90.75 ± 2.6	83.42 ± 2.6	ns
	(*n* = 12)	(*n* = 12)	
Mount frequency	14.00 ± 0.7	14.00 ± 0.7	ns
	(*n* = 12)	(*n* = 12)	
Intromission latency (s)	181.17 ± 5.2	161.75 ± 5.2	*
	(*n* = 12)	(*n* = 12)	
Intromission frequency	13.33 ± 0.6	10.42 ± 0.6	*
	(*n* = 12)	(*n* = 12)	
Ejaculation latency (s)	130.25 ± 6.6	154.08 ± 6.6	*
	(*n* = 12)	(*n* = 12)	
Ejaculation frequency	2.25 ± 0.1	1.98 ± 0.1	ns
	(*n* = 12)	(*n* = 12)	
Post-ejaculation interval (s)	172.83 ± 5.3	154.67 ± 5.3	*
	(*n* = 12)	(*n* = 12)	

SE: Standard error of the mean, s: Second, ns: Not significant, * significant at *p* < 0.05.

**Table 7:** Reproductive performance of F1 male rat mated with non-treated females in each group.

Variables	Groups	
	C	WR
	Mean ± SE	Mean ± SE
Body weight of non-treated dams at birth (g)	227.56 ± 4.2	228.21 ± 4.4
	(*n* = 16)	(*n* = 14)
Litter weight of F2 at birth (g)	53.86 ± 2.7	56.75 ± 2.8
	(*n* = 15)	(*n* = 14)
Individual litter weight of F2 at birth (g)	5.50 ± 0.1	5.70 ± 0.1
	(*n* = 15)	(*n* = 14)
Litter size of F2 at birth (pup)	9.86 ± 0.5	10.00 ± 0.5
	(*n* = 15)	(*n* = 14)

SE: Standard error of the mean.
